# N-Doped Graphene as an Efficient Metal-Free Electrocatalyst for Indirect Nitrate Reduction Reaction

**DOI:** 10.3390/nano11092418

**Published:** 2021-09-17

**Authors:** Jujiao Zhao, Bo Shang, Jun Zhai

**Affiliations:** 1 College of Environment and Ecology & MOE Key Laboratory of the Three Gorges Reservoir Region’s Eco-Environment, Chongqing University, Chongqing 400045, China; zhaojujiao@cqu.edu.cn; 2 School of Chemistry and Chemical Engineering & Chongqing Key Laboratory of Theoretical and Computational Chemistry, Chongqing University, Chongqing 401331, China; bshang@cqu.edu.cn

**Keywords:** nitrate reduction reaction, N-doped graphene, pyridinic-N

## Abstract

N-doped graphene samples with different N species contents were prepared by a two-step synthesis method and evaluated as electrocatalysts for the nitrate reduction reaction (NORR) for the first time. In an acidic solution with a saturated calomel electrode as reference, the pyridinic-N dominant sample (NGR2) had an onset of 0.932 V and a half-wave potential of 0.833 V, showing the superior activity towards the NORR compared to the pyrrolic-N dominant N-doped graphene (onset potential: 0.850 V, half-wave potential: 0.732 V) and the pure graphene (onset potential: 0.698 V, half-wave potential: 0.506 V). N doping could significantly boost the NORR performance of N-doped graphene, especially the contribution of pyridinic-N. Density functional theory calculation revealed the pyridinic-N facilitated the desorption of NO, which was kinetically involved in the process of the NORR. The findings of this work would be valuable for the development of metal-free NORR electrocatalysts.

## 1. Introduction

The global nitrogen cycle has been disrupted by human activities and industries, resulting in an increased nitrate concentration in the hydrosphere [1,2]. Since a high nitrate level in the drinking water has been proven to be one of the inducements of methemoglobinemia and gastrointestinal cancer, it is important to limit the concentration of nitrate in groundwater and other water bodies [3,4,5]. Reducing the nitrate emission from industry effluents is an effective way to limit nitrate pollution since nitric acid is widely used in several industries, such as the nuclear industry and the explosives industry [6,7]. The wastewater from these industries could be strongly acidic with a high concentration of nitrate, which is hardly treated by traditional biological treatments [7]. An efficient method of removing nitrate from acidic solutions is highly needed.

The electrochemical reduction of nitrate has attracted huge interest thanks to its controllable process and relatively low costs [8]. However, the sluggish kinetics of the nitrate reduction reaction (NORR) leads to an extreme demand for robust electrocatalysts [9,10,11]. Generally, noble metals, such as Pt [12,13,14,15,16,17], Rh [18,19], Au [20], and Pd [21,22,23] based catalysts, present excellent electrochemical activity for the NORR, but the scarcity and high cost hinder their practical applications. To develop cheaper alternatives, most efforts have been devoted into metal-based electrodes, such as Cu [24,25,26,27,28], Sn [29,30,31], Co [32], Fe [33], and Cu-based bimetallic metals (Cu-Zn, Cu-Ni, Cu-Mn, etc.) [34,35,36]. However, the possible release of metal ions during operation also limits their usefulness for practical applications, especially under acidic conditions. Despite many efforts devoted to developing electrocatalysts for the NORR, few of these studies involved the NORR under strongly acidic conditions [8]. By comparison, cheap and earth-abundant metal-free electrocatalysts with high activity and good stability in acidic conditions are highly desired.

Carbon nanomaterial is widely used as electrocatalysts due to their chemical stability and good conductivity [37,38,39,40,41,42]. Graphene, the typical carbon nanomaterial with two-dimensional structure, has been demonstrated to facilitate the electrochemical reduction of nitrate into nitrite [43], which is regarded as the rate-determining step in the entire process of nitrate reduction [44,45,46,47]. The NORR on graphene is effective in acidic solution through the indirect process with NO as the key intermediate. According to the literature, the indirect NORR on graphene even presented better performance than that on Pt [43]. However, there are still scarce reports on the NORR with graphene-based catalysts. It is still highly desirable to develop carbon-based metal-free electrocatalysts with higher NORR performance than pristine graphene.

N doping can significantly enhance the electrochemical activity of graphene for oxygen reduction [48], oxygen evolution [49], hydrogen evolution [50], and CO_2_ electroreduction [51], due to the change of charge densities. The improved charge densities of surrounding C atoms may facilitate the electron-transfer reaction, which also suggests an improved activity for the NORR. However, to the best of our knowledge, no related study has been reported. Furthermore, since the effects of N species (pyridinic-N, pyrrolic-N, and graphitic-N) on the electrochemical activity are substantially different [52,53], it would be meaningful to reveal their contribution to the NORR, which will guide the design of superior metal-free electrocatalysts for the NORR in the future. 

Herein, we synthesized N-doped graphene containing different N species and evaluated their NORR activity. For the first time, an N-doped carbon material was adopted as the NORR electrocatalyst and the contribution of active N species was investigated. We demonstrated that pyridinic-N played the dominant role in the enhanced NORR activity and density functional theory (DFT) calculations were further conducted to reveal the mechanism.

## 2. Materials and Methods

### 2.1. Materials

All solid chemicals and 20% commercial Pt/C were purchased from Aladin Ltd. (Shanghai, China). HNO_3_, HCl, H_2_O_2_, and H_2_SO_4_ were obtained from Chongqing Chuandong Co., Ltd. (Chongqing, China). Dupont Nafion PFSA Polymer Dispersion D-520 was obtained from Dupont. 

### 2.2. Preparation of N-Doped Graphene

Firstly, graphene oxide (GO) was prepared by a modified Hummer’s method [52]. Then, GO was reduced by hydrothermal method to produce graphene. Urea or melamine was chosen as precursor to provide N atoms during the hydrothermal reaction. In a typical process, 35 mg of GO and 1050 mg of urea were mixed with 35 mL of de-ionized (DI) water and transferred into a Teflon-lined stainless-steel autoclave. After heating at 180 °C for 12 h and then cooling down to room temperature, the as-prepared sample was washed by DI water and dried overnight to obtain the N-doped graphene named as NGR1. NGR2 was produced by a similar hydrothermal process but with 35 mg of GO and 800 mg of melamine as precursors. Pure reduced GO (GR) was also prepared by a similar hydrothermal process without the addition of N-containing precursors.

### 2.3. Characterizations

Scanning electron microscopy (SEM, Hitachi S-4800) and transmission electron microscopy (TEM, FEI-Tecnai G2 20) were used to characterize the morphology of catalysts. Energy dispersive X-ray spectrometer (EDS) elemental mapping was recorded on an Iridium Ultra Premium EDS System (A550I, IXRF, USA). X-ray diffraction (XRD) patterns were carried out on a Shimadzu LabX XRD-6000 to investigate the crystal structure. X-ray photoelectron spectra (XPS) were obtained with an ESCALAB 250Xi spectrometer using a nonmonochromatized A1 Kα X-ray source (1486.6 eV) for elemental composition analyses, together with a Renishaw Micro-Raman system 2000 with He-Ne laser excitation for Raman spectroscopy. N_2_ adsorption–desorption isotherms were obtained at 77 K with a Quadrasorb 2MP instrument. 

### 2.4. Electrochemical Performance for the NORR

Electrochemical measurements were performed on a CHI660E electrochemical workstation in a three-electrode cell. The saturated calomel electrode (SCE) and Pt wire were chosen as reference electrode and counter electrode, respectively. All the potentials mentioned in this work are versus SCE. For the electrode preparation, the prepared catalyst powders were mixed with a Nafion solution and loaded on glassy carbon with a mass loading of 0.2 mg cm^−2^ for all the samples. Linear sweep voltammetry was carried out in a 5 M HNO_3_ solution with a scan rate of 10 mV s^−1^. Chronoamperometry was conducted in a 5 M H_2_SO_4_ solution at 0.8 V in the absence and presence of 2 mM HNO_3_. The nitrate removal experiment was conducted in a two-chamber reactor divided by a Nafion 117 membrane. The working electrode was prepared with a carbon cloth as the substrate with an area of 4 cm^2^. The volume of the solution was 20 mL. The concentrations of nitrate and nitrite were determined by the standard colorimetric method according to the Chinese Environment Standards using a UV-2450 spectrophotometer (Text S2).

### 2.5. DFT Calculation Details

The Vienna Ab initio Package (VASP) [54,55] was employed to perform all the DFT calculations within the generalized gradient approximation (GGA) using the PBE formulation (details were described in Text S1) [56]. Three graphene models corresponding to the undoped graphene with defects (model 1), graphene with pyridinic-N (model 2), and graphene with pyrrolic-N (model 3) were constructed. The adsorption energy (E_ads_) of NO was defined as:

E_ads_ = E_NO/surf_ − E_surf_ − E_NO (g)_ (1)

where E_NO/surf_, E_surf_, and E_NO (g)_ are the energy of NO adsorbed on the surface, the energy of clean surface, and the energy of isolated NO molecules in a cubic periodic box with a side length of 20.00 Å and a 1 × 1 × 1 Monkhorst–Pack k-point grid for Brillouin zone sampling, respectively.

## 3. Results and Discussions

### 3.1. Characterizations of Samples

**Figure 1:**
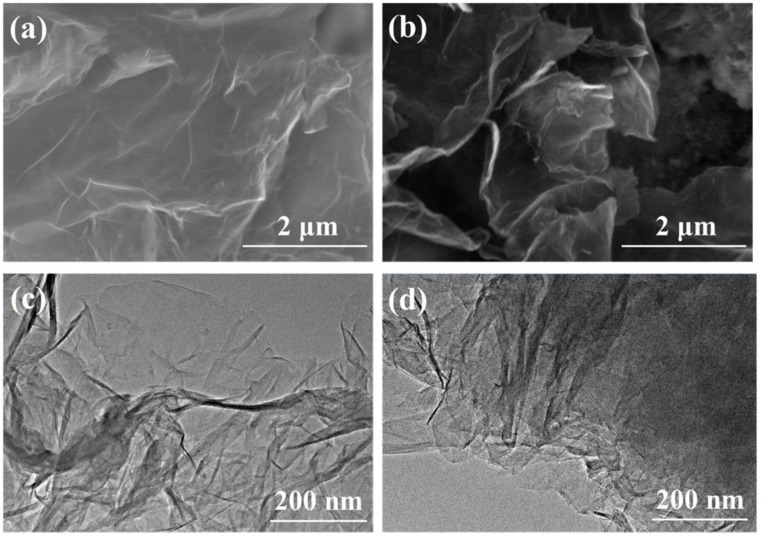
(**a**,**b**) SEM and (**c**,**d**) TEM images of NGR1 and NGR2.

To accurately investigate the effects of different N species on the NORR performance, NGR1 and NGR2 with similar elemental contents (Table S1) were prepared under similar hydrothermal conditions by controlling the N-containing precursors. Figure 1a,b shows the morphology of NGR1 and NGR2. Both samples exhibited the typical two-dimensional structure of graphene with massive wrinkles. No residues could be found on the graphene surface, indicating the samples consisted of only graphene without any undecomposed precursors. TEM (Figure 1c,d) was also employed to reveal the microstructure of the samples, which further indicated NGR1 and NGR2 possessed a voile-like topography. From the EDS images (Figures S1 and S2), the color plots representing the N atoms were dispersed uniformly throughout the selected sample area, indicating the successful doping of N into the graphene.

**Figure 2:**
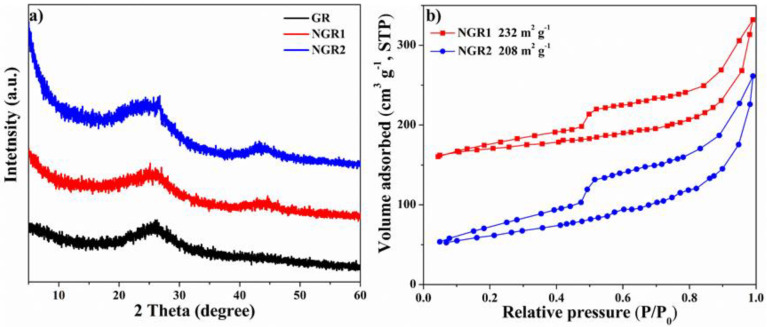
(**a**) XRD patterns of NGR1, NGR2, and GR, and (**b**) N_2_ adsorption–desorption curves of NGR1 and NGR2.

X-ray diffraction (XRD) patterns of NGR1, NGR2, and GR (Figure 2a) exhibited similar broad peaks centered at 20°~30° corresponding to the carbon (002) facet. The absence of peak relating to graphene oxide at 10° in all three patterns indicated that the graphene was successfully prepared by reducing the graphene oxide through hydrothermal method. The Brunauer–Emmett–Teller (BET) surface areas of NGR1 and NGR2 were revealed by N_2_ adsorption–desorption isotherms, as shown in Figure 2b. NGR1 and NGR2 had surface areas of 232 and 208 m^2^ g^−1^, respectively. It is known that the BET surface area significantly affects electrochemical activity. The difference in BET surface areas between NGR1 and NGR2 was less than 10%, which is helpful to rule out the influence of the BET surface area on the NORR activity.

**Figure 3:**
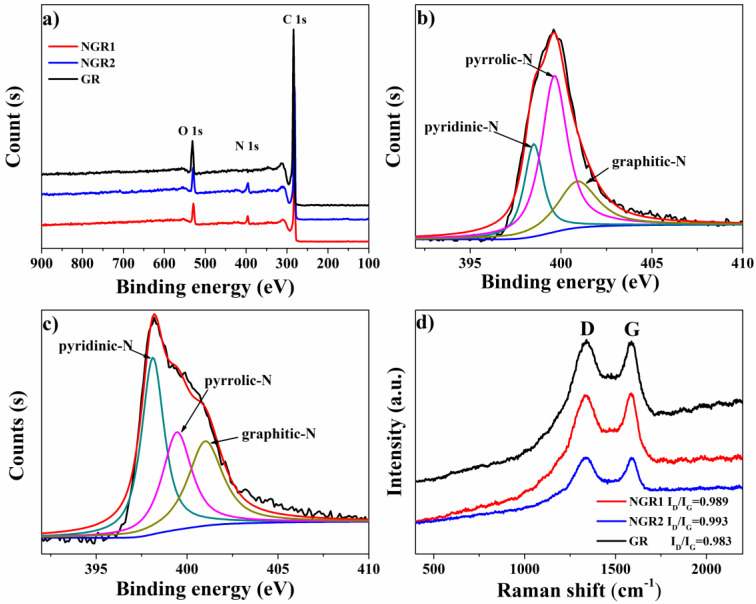
(**a**) Full survey XPS spectra of NGR1, NGR2, and GR. High-resolution N 1s spectra of NGR1 (**b**) and NGR2 (**c**), respectively; (**d**) Raman spectra of GR, NGR1, and NGR2.

XPS was employed to reveal the elemental composition of samples. As shown in Figure 3a, compared to the GR without adding N-containing precursor in synthetic process, the full survey XPS spectra of NGR1 and NGR2 presented obvious peaks corresponding to C 1s, O 1s and N 1s, further indicating the successful N doping in samples. Table S1 indicates that the N contents of NGR1 and NGR2 were 5.35% and 5.19%, respectively. The high-resolution N 1s spectra of both NGR1 and NGR2 (Figure 3b,c) could be deconvoluted into three peaks located at 398.1 eV (pyridinic-N), 399.5 eV (pyrrolic-N) and 401.1 eV (graphitic-N), respectively [52]. However, it is worth noting that pyrrolic-N was the dominant N species in NGR1 and its content reached 50.9% of total N. In comparison, NGR2 had a higher content of pyridinic-N (40.48% of total N) over pyrrolic-N. In addition, the graphitic-N contents of NGR1 and NGR2 were similar. This difference could be attributed to the different structures of N precursors for synthesizing the samples (urea for NGR1 and melamine for NGR2). Based on these analyses, the different N-species-dominated NGR was synthesized.

Since it was reported that graphene defects could be the active sites for the NORR, it was important to clarify the degree of structural defect of the samples before evaluating the effect of N doping [43]. Raman spectroscopy was employed here and the spectra of all samples presented two prominent peaks. The peak located at 1350 cm^−1^ corresponded to the D band, which was caused by the disorder in graphitic structure of carbon materials, and the G band peak, located at 1580 cm^−1^, arose from the sp^2^ hybridized graphitic carbon atoms. Generally, the D/G peak intensity ratio (I_D_/I_G_) was highly correlated with the quantity of structural defects, which may reveal the degree of structural defect. As shown in Figure 3d, the I_D_/I_G_ values for NGR1, NGR2, and GR were 0.989, 0.993, and 0.983, respectively, indicating the three samples had a similar degree of structural defect. Therefore, the effect of N species, i.e., pyrrolic-N and pyridinic-N, on the electrochemical performance could be accessible. 

### 3.2. Electrochemical Measurements of N-Doped Samples

**Figure 4:**
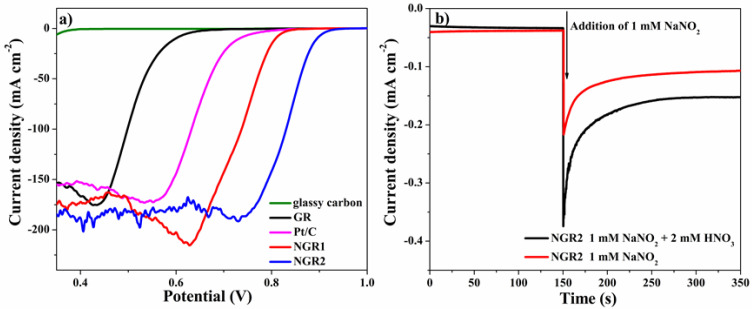
(**a**) Linear sweep voltammetries of glass carbon, GR, Pt/C, NGR1, and NGR2 in 5 M HNO_3_ solution with a scan rate of 10 mV s^−1^. (**b**) Chronoamperometric response of NGR2 in 5 M H_2_SO_4_ at 0.8 V in the absence (red line) and presence (black line) of 2 mM HNO_3_. A total of 1 mM NaNO_2_ was dropped into the solution at 150 s.

Linear sweep voltammetries (LSVs) were measured in a 5 M HNO_3_ solution with a scan rate of 10 mV s^−1^ to investigate the NORR activity of the samples. Each sample was tested in a fresh solution to exclude the possible by-products produced during the survey. As shown in Figure 4a, the glassy carbon electrode without any electrocatalyst showed negligible current response, which indicated that glassy carbon electrode, the substrate for all samples, had a poor catalytic activity for the NORR. After loading with GR, it presented an onset potential of 0.698 V (in this work, the onset potential was defined as the potential to deliver a current density of 1 mA cm^−2^) and a half-wave potential of 0.506 V, indicating that GR was electrochemical active towards the NORR. However, the activity of GR was still lower than that of commercial 20 wt % Pt/C (Pt/C) since Pt/C had a higher onset potential (0.83 V) and half-wave potential (0.642 V). When NGR1 was adopted, the onset potential and the half-wave potential positively shifted to 0.85 V and 0.732 V, respectively, which were 20 mV and 90 mV more positive, respectively, than that of Pt/C, demonstrating the higher activity of NGR1 compared to Pt/C. Since NGR1 and GR had the same degree of structural defect revealed by Raman measurements, the significantly enhanced activity of NGR1 could be attributed to the N doping. 

To further investigate the effect of different N species on the NORR performance, the activity of pyridinic-N-dominated NGR2 was also tested. The onset potential of NGR2 further increased to 0.932 V, which was 83 mV more positive than that of NGR1. Additionally, the half-wave potential of NGR2 was 101 mV more positive than that of NGR1. Although the catalytic activity of pyridinic- or pyrrolic-N sites alone could not be distinguished based on our experimental results, the boosted electrochemical performance of NGR2 compared to NGR1 implied that pyridinic-N played an important role in the enhanced NORR activity of N-doped graphene since the major difference between NGR1 and NGR2 was the contents of pyridinic-N and pyrrolic-N.

### 3.3. Investigation into the Mechanism of Electrochemical NORR on N-Doped Graphene

The mechanism of the NORR on N-doped graphene was further discussed. Kamiya et al. [43] reported that the autocatalytic mechanism (Equations (2)–(5)) on pure graphene was similar to that on Pt [45] and that graphene defects could facilitate the adsorption of NO^+^ to enhance the activity, since the C atoms located at defects had different density states. The N doping could change the charge densities of surrounding C atoms due to the larger electronegativity of N (3.04) compared to C (2.55), therefore we speculated that the mechanism for the NORR on N-doped graphene might be the same.

2 NO^+^ + 2e^−^ → 2 NO (2)

2 NO + HNO_3_ + H_2_O ⇋ 3 HNO_2_ (3)

2 HNO_2_ + 2 H^+^ ⇋ 2 NO^+^ + 2 H_2_O (4)

HNO_3_ + 2 H^+^ + 2 e^−^ → HNO_2_ + H_2_O (overall reaction) (5)

According to this mechanism, the nitrite was not only the product, but also a key intermediate, since the nitrite could be reduced into NO^+^, as shown in Equation (4). To demonstrate the critical role of nitrite for the reduction of nitrate in the autocatalytic reaction, a chronoamperometric measurement was employed here with NGR2 in a 5 M H_2_SO_4_ solution in the absence or presence of 2 mM nitrate at 0.8 V, which was more positive than the onset potential of GR. As shown in Figure 4b, the NGR2 in the presence of 2 mM nitrate presented no current response towards the NORR compared to NGR2 in the absence of 2 mM nitrate. After adding 1 mM NaNO_2_, NGR2 with NaNO_2_ presented a reduction current corresponding to the electrochemical reduction of nitrite. Remarkably, NGR2 with both 2 mM nitrate and 1 mM NaNO_2_ exhibited a larger current than that of NGR2 with only 1 mM NaNO_2_. The larger current could be ascribed to the sum of the reduction currents of nitrite and nitrate, indicating that the nitrate could be reduced in the presence of nitrite. The results demonstrated that nitrite was the important reactive intermediate in the NORR.

The nitrate removal experiment with NGR2 as the working electrode was conducted in the presence and absence of nitrite to identify the electrochemical reduction of nitrate, as well as provide more evidence supporting the importance of nitrite in this process. A two-chamber reactor was employed to exclude the influence of the counter electrode. The initial concentration of nitrate was 10 mM and the electrolyte was 5 M H_2_SO_4_. As shown in Figure S3a, only 4.4% of nitrate was removed within 180 min at the potential of 0.6 V without the addition of nitrite. In the presence of 10 mM nitrite at the onset, 30.4% of nitrate was removed within 180 min, further demonstrating the important role of nitrite in the indirect NORR on NGR2. It could be noticed that the removal kinetics decreased after 90 min. The concentration of nitrite was also detected, as shown in Figure S3b. The decreased kinetics could be attributed to the decreased concentration of nitrite, which is also reduced by the electrode under this condition.

**Figure 5:**
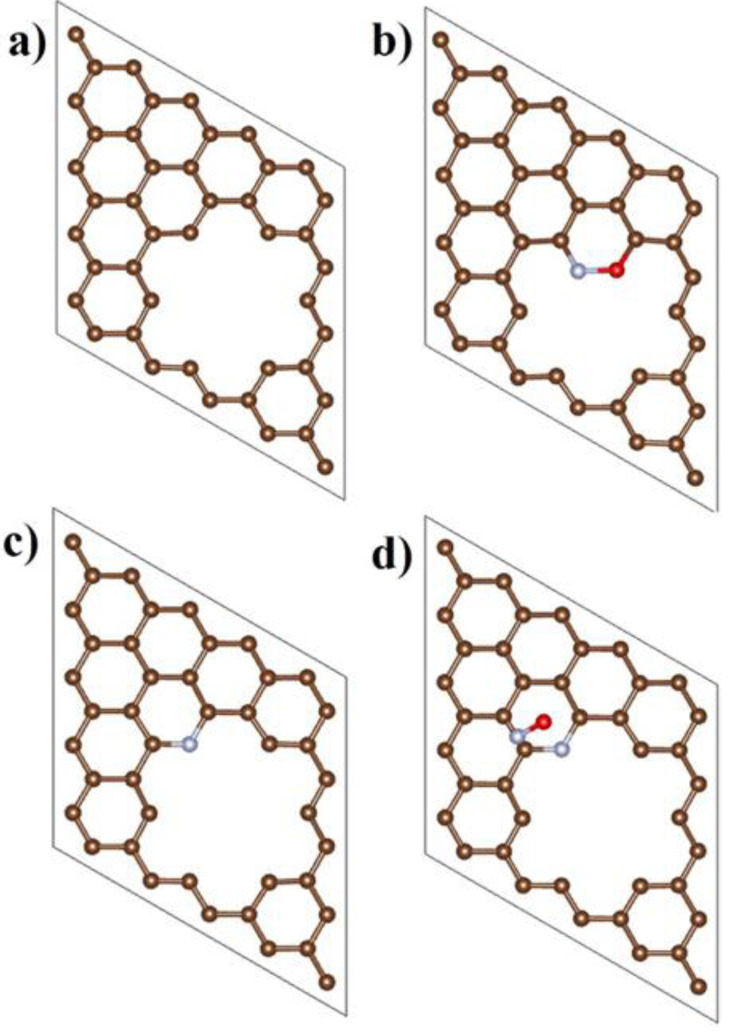
Top view of the modeling of (**a**) the undoped graphene and (**c**) graphene with pyridinic-N with defects; Adsorption of NO on (**b**) undoped graphene and (**d**) on graphene with pyridinic-N after optimization.

To further reveal the effect of N doping on the NORR activity, DFT calculations were performed. The enhanced adsorption of NO^+^ onto the edge of graphene rather than the basal plane of graphene has been reported by Kamiya et al. [43]. Due to the positive charge of NO^+^, the adsorption of NO^+^ on the edge of graphene was extremely strong. It is well known that N doping results in the presence of defects on the graphene. Thus, it was reasonable to speculate that Equation (2) was not the rate-determining step. Based on the proposed mechanism, another important process was related to the desorption of NO according to Equation (3). NO was expected to be desorbed from the active sites so that it could react with HNO_3_ to produce HNO_2_ and the active sites were therefore regenerated for the next reactive cycle. By taking these into consideration, the adsorption energies of NO (E_ads_) on different types of graphene were calculated and are shown in Figure 5. Since it was reported that the C atom on the edge of graphene was the active site for the NORR [43] and the C atom near the N atom was influenced by the N doping mostly [57,58], the C atom near the N atom was treated as the active site and the desorption of NO was calculated. E_ads_ for undoped graphene with defects was −5.89 eV meanwhile the E_ads_ for graphene with pyridinic-N was only −0.12 eV. The more positive E_ads_ of NO on graphene with pyridinic-N than those of NO on undoped graphene with defects, indicated that NO would drop from the graphene with pyridinic-N more easily. These results demonstrate that the pyridinic-N doping facilitated the desorption of NO as well as the regeneration of active sites. The adsorption of NO on the graphene with pyrrolic-N was also calculated but the result after optimization was unreasonable, implying that the C atom near pyrrolic-N atom was not the active site. Considering pyridinic-N existed in both of NGR1 and NGR2 but with 1.8 times higher content in NGR2, the excellent NORR performance of NGR2 compared to NGR1 implied a positive correlation between the content of pyridinic-N and the NORR activity. Thus, both the experimental results and DFT calculations indicated that pyridinic-N played the most important role in the NORR and that the facilitated desorption of NO might be a possible origin of the enhanced NORR performance of N-doped graphene.

## 4. Conclusions

In summary, we prepared N-doped graphene and evaluated its electrochemical performance towards the NORR in an acidic solution. To the best of our knowledge, it was the first time that N doping carbon materials were adopted as NORR electrocatalysts. The N doping could significantly improve the electrocatalytic activity of graphene for the NORR, which was better than the activity of commercial Pt/C. Furthermore, our results indicated that Pyridinic-N played an important role in the NORR by facilitating the desorption of NO from reaction sites as shown by density functional theory calculations. The findings of this work shed lights on the new application of N doping carbon-based materials and would be valuable for the design of metal-free electrocatalyst for the NORR.

## Supplementary Materials

The following are available online at https://www.mdpi.com/article/10.3390/nano11092418/s1, Figure S1: (a) SEM and (b–d) EDS mapping of C, O, and N in NGR1, respectively, Figure S2: (a) SEM and (b-d) EDS mapping of C, O, and N in NGR2, respectively, Figure S3: (a) Electrochemical reduction of NO_3_^−^ on NGR2 at 0.6 V for 3 h, with and without initial 10 mM NO_2_^−^, with a 5 M H_2_SO_4_ electrolyte; (b) the amount of NO_2_^−^ on NGR2 at 0.6 V for 3 h with the initial 10 mM NO_2_^−^ and 10 mM NO_3_^−^, Table S1: Summary of chemical composition analysis from XPS and Raman results and electrochemical properties of samples, Text S1: Computational details of the DFT calculations, Text S2: Detection of nitrate and nitrite.
