# Supplementary material for: N-Doped Graphene as an Efficient Metal-Free Electrocatalyst for Indirect Nitrate Reduction Reaction
Source: Nanomaterials (Basel). 2021 Sep 17;11(9):2418. doi: 10.3390/nano11092418 (PMC8470669; doi:10.3390/nano11092418)
Supplement: Supplementary file 1 [file nanomaterials-11-02418-s001.zip › nanomaterials-1363257-supplementary.pdf]

Supporting Information

N-Doped Graphene as an Efficient Metal-Free Electrocatalyst for Indirect Nitrate Reduction Reaction

Jujiao Zhao [1], Bo Shang [2] and Jun Zhai [1,*]

[1] College of Environment and Ecology & MOE Key Laboratory of the Three Gorges Reservoir
Region's Eco-Environment, Chongqing University, Chongqing 400045, China; zhaojujiao@cqu.edu.cn

[2] School of Chemistry and Chemical Engineering & Chongqing Key Laboratory of Theoretical
and Computational Chemistry, Chongqing University, Chongqing 401331, China; bshang@cqu.edu.cn

* Correspondence: zhaijun@cqu.edu.cn

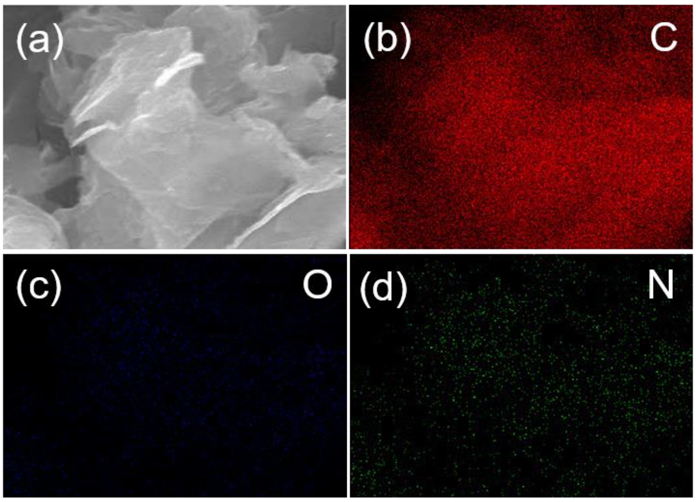

Figure S1. (a) SEM and (b-d) EDS mapping of C, O, and N in NGR1, respectively.

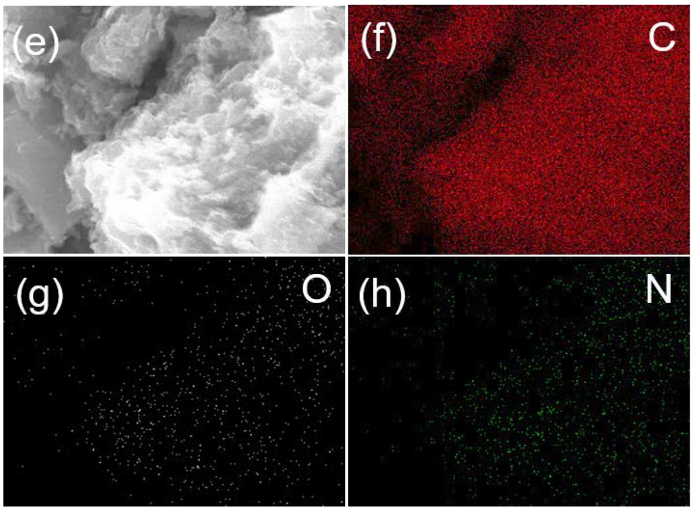

Figure S2. (a) SEM (b-d) EDS mapping of C, O, and N in NGR2, respectively.

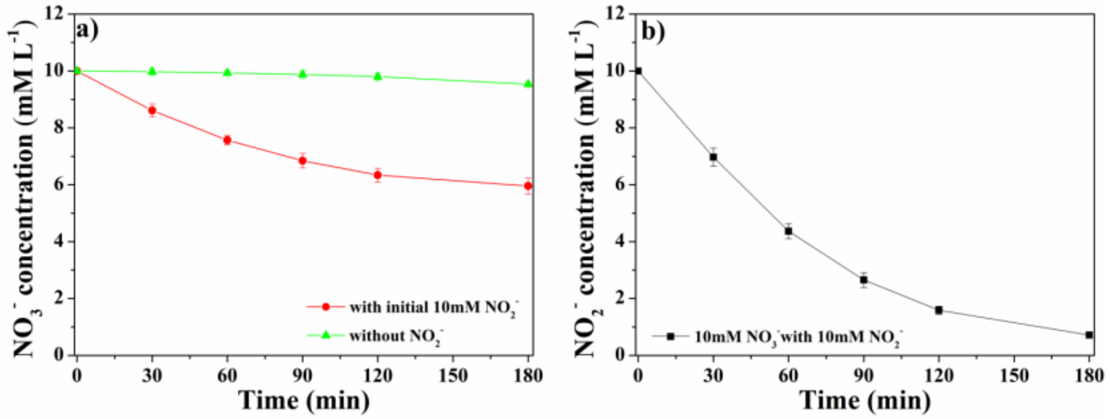

Figure S3. (a) Electrochemical reduction of NO_3^- on NGR2 at 0.6 V for 3 h, with and without initial 10 mM NO_2^- with a 5 M H_2SO_4 electrolyte; (b), amount of NO_2^- on NGR2 at 0.6 V for 3 h with the initial 10 mM NO_2^- and 10 mM NO_3^-.

Table S1 Summary of chemical composition analysis from XPS and Raman results and electrochemical properties of samples.

**Sample**	**At% (C)**	**At% (O)**	**At% (N)**	**Pyridinic-N of total N (%)**	**Pyrrolic-N of total N (%)**	**Graphitic-N of total N (%)**	**I_D/I_G**	**Onset Potential (V)**	**Half-wave Potential (V)**
GR	84.56	15.35	n/a	n/a	n/a	n/a	0.983	0.698	0.506
NGR1	86.26	8.39	5.35	23	50.9	26.1	0.989	0.85	0.732
NGR2	86.81	8	5.19	40.48	29.34	30.18	0.993	0.932	0.833

Text S1 Computational details of the DFT calculations

The calculations were performed using spin-polarized DFT as implemented in VASP. We chose the projected augmented wave (PAW) potentials [1,2] to describe the ionic cores and took valence electrons into account using a plane wave basis set with a kinetic energy cutoff of 400 eV. Partial occupancies of the Kohn−Sham orbitals were allowed using the Gaussian smearing method and a width of 0.05 eV. The electronic energy was considered self-consistent when the energy change was smaller than 10−5 eV. A geometry optimization was considered convergent when the force change was smaller than 0.02 eV/Å. Grimme's DFT-D3 methodology [3] was used to describe the dispersion interactions.

The equilibrium lattice constant of hexagonal graphene unit cell separated by a vacuum layer in the depth of 20.00 Å was optimized to be a=2.468 Å, when using a 15×15×1 Monkhorst−-Pack k-point grid for Brillouin zone sampling. We then used it to construct a graphene sheet model with (5×5) periodicity in the x and y directions and 1 atomic layer in the z direction by a vacuum layer in the depth of 15 Å in order to separate the surface slab from its periodic duplicates. This graphene sheet model contained 50 C atoms. We constructed three graphene models. In model 1, six neighboring C atoms were removed from this model in order to mimic the vacant graphene. In model 2, one C atom on the edge of model 1 was replaced by N in order to mimic pyridinic-N on graphene. In model 3, one N atom was added to the edge of model 1 in order to mimic pyrrolic-N on graphene. During structural optimizations, the gamma point in the Brillouin zone was used for k-point sampling, and all atoms in the models were allowed to relax.

References

[1] Kresse, G.; Joubert, D. From Ultrasoft Pseudopotentials to the Projector Augmented-Wave Method. Phys. Rev. B 1999, 59, 1758-1775.

[2] Blöchl, P. E. Projector Augmented-Wave Method. Phys. Rev. B 1994, 50, 17953−17979.

[3] Grimme, S.; Antony, J.; Ehrlich, S.; Krieg, H. J. Chem. Phys. 2010, 132, 154104.

Text S2 Detection of nitrate and nitrite

Nitrate: National Environmental Protection Standard of the People's Republic of China, Water Quality - Determination of Nitrate-nitrogen - Ultraviolet spectrophotometry, HJ/T 346-2007.
Nitrite: National Standard of the People's Republic of China, Water Quality - Determination of nitrogen (nitrite) - Spectrophotometric method, GB 7489-87.
